# Exercise Mitigates the Loss of Muscle Mass by Attenuating the Activation of Autophagy during Severe Energy Deficit

**DOI:** 10.3390/nu11112824

**Published:** 2019-11-19

**Authors:** Marcos Martin-Rincon, Alberto Pérez-López, David Morales-Alamo, Ismael Perez-Suarez, Pedro de Pablos-Velasco, Mario Perez-Valera, Sergio Perez-Regalado, Miriam Martinez-Canton, Miriam Gelabert-Rebato, Julian William Juan-Habib, Hans-Christer Holmberg, Jose A L Calbet

**Affiliations:** 1 Department of Physical Education and Research Institute of Biomedical and Health Sciences (IUIBS), University of Las Palmas de Gran Canaria, Campus Universitario de Tafira, s/n, 35017 Las Palmas de Gran Canaria, Canary Islands, Spain; marcos.martinrincon@gmail.com (M.M.-R.); moralesalamo.d@gmail.com (D.M.-A.); ismaelperezsuarez@gmail.com (I.P.-S.); marioperezvalera@gmail.com (M.P.-V.); serperel284@gmail.com (S.P.-R.); martinezcantonmiriam@gmail.com (M.M.-C.); miriamgela@hotmail.com (M.G.-R.);; 2 Department of Biomedical Sciences, Faculty of Medicine and Health Sciences, University of Alcalá, Ctra. Madrid-Barcelona, km 33,600, 28871 Alcalá de Henares, Madrid, Spain; alberto_perez-lopez@hotmail.com; 3 Department of Endocrinology and Nutrition, Hospital Universitario de Gran Canaria Doctor Negrín, Calle Plaza Barranco de la Ballena, s/n, 35010 Las Palmas de Gran Canaria, Canary Islands, Spain; 4 Department of Health Sciences, Mid Sweden University, SE-83140 Östersund, Sweden; hans-christer.holmberg@miun.se; 5 School of Kinesiology, Faculty of Education, The University of British Columbia, Vancouver, BC V6T 1Z, Canada; 6 Department of Physical Performance, The Norwegian School of Sport Sciences, Postboks, 4014 Ulleval Stadion, 0806 Oslo, Norway

**Keywords:** autophagy-lysosome, caloric restriction, protein degradation, skeletal muscle, ubiquitin-proteasome

## Abstract

The loss of skeletal muscle mass with energy deficit is thought to be due to protein breakdown by the autophagy-lysosome and the ubiquitin-proteasome systems. We studied the main signaling pathways through which exercise can attenuate the loss of muscle mass during severe energy deficit (5500 kcal/day). Overweight men followed four days of caloric restriction (3.2 kcal/kg body weight day) and prolonged exercise (45 min of one-arm cranking and 8 h walking/day), and three days of control diet and restricted exercise, with an intra-subject design including biopsies from muscles submitted to distinct exercise volumes. Gene expression and signaling data indicate that the main catabolic pathway activated during severe energy deficit in skeletal muscle is the autophagy-lysosome pathway, without apparent activation of the ubiquitin-proteasome pathway. Markers of autophagy induction and flux were reduced by exercise primarily in the muscle submitted to an exceptional exercise volume. Changes in signaling are associated with those in circulating cortisol, testosterone, cortisol/testosterone ratio, insulin, BCAA, and leucine. We conclude that exercise mitigates the loss of muscle mass by attenuating autophagy activation, blunting the phosphorylation of AMPK/ULK1/Beclin1, and leading to p62/SQSTM1 accumulation. This includes the possibility of inhibiting autophagy as a mechanism to counteract muscle loss in humans under severe energy deficit.

## 1. Introduction

When the energy balance is negative, as during dieting for weight loss or in several conditions in patients, ∼25% to 40% of the total reduction in weight is due to loss of skeletal muscle [1,2]. In critical patients, the loss of muscle mass is associated with unfavorable clinical outcomes [3,4]. In obese and overweight, an excessive loss of fat-free mass (FFM) may also be detrimental since skeletal muscle accounts for a significant fraction of the resting metabolic rate, and is essential for exercising and metabolic health [5]. Accordingly, there is considerable interest in minimizing the loss of FFM in people dieting and in admitted patients with energy deficit [6]. Exercise and increasing the ratio of protein-to-carbohydrate in the diet attenuate the loss of FFM during mild-to-moderate energy deficits when dieting [7], but the underlying molecular mechanisms remain largely unknown. In patients with septic shock, a mild exercise program halved the loss of muscle mass and was associated with an attenuation of an excessive activation of autophagy [6]. However, multiple administration of drugs, including insulin, limits the interpretations of these results [6]. Moreover, it remains unknown what is the optimal amount of exercise needed to prevent the loss of muscle mass caused by a negative energy balance.

A negative energy balance elicits an overall increase in whole-body proteolysis, amino acid oxidation, and nitrogen excretion [8,9,10], accompanied by a reduction in muscle protein synthesis [11]. Compared to muscle protein synthesis, little attention has been given to the molecular mechanisms driving the muscle proteolytic response to energy deficit and its interaction with exercise [6,12,13,14], and no study to date has addressed the molecular regulation of protein degradation during extreme energy deficits in human skeletal muscle.

Protein degradation in skeletal muscle primarily depends on the activation of the two major proteolytic pathways, the ubiquitin-proteasome system (UPS) and the autophagy-lysosome system (ALS). These two systems are coordinated by the members of the forkhead box O family of transcription factors (FoxOs), particularly by FoxO1and FoxO3a [15].

The UPS requires ubiquitin ligase enzymes (E3s) to catalyze the attachment of polyubiquitin chains to the protein substrate, which then is targeted to the 26S proteasome for degradation. Among the E3s, the gene expression of muscle ring finger 1 (MuRF1) and muscle atrophy F box (MAFbx or Atrogin 1) is upregulated in animal and human models of muscle atrophy [16,17,18], although human data are scarce and less consistent [3,4,6]. One of the proteins targeted by MAFbx for subsequent proteasome degradation during protein breakdown is the eukaryotic translation initiation factor 3 subunit f (eIF3f), which plays a pivotal role in the regulation of muscle mass [19]. Protein degradation through the ALS is required for muscle mass maintenance [20] and a physiological adaptation to regular exercise [21,22,23]. Adenosine monophosphate-activated protein kinase (AMPK) activates autophagy and is necessary for exercise-mediated autophagy induction [24,25]. The formation of the autophagosome is elicited by the unc-51-like kinase 1 (ULK1), which is activated and inhibited by AMPK and mTOR, respectively [26]. ULK1 activates Beclin 1 through its phosphorylation on Ser15, resulting in Beclin 1-Vps34 (vacuolar sorting protein 34) complex activation, a step necessary for complete autophagy induction [27]. Nucleation and expansion of the autophagosome require microtubule-associated protein 1 light chain 3 beta I (LC3BI) lipidation to LC3BII, which is recruited to the autophagosomal membrane [28]. Ubiquitinated proteins and LC3BII bind to the sequestosome 1 (p62/SQSTM1) for degradation together with the autophagosome content following fusion with the lysosome [28].

Studies on short-term energy deficit have found little or no change in gene and protein expression markers of protein breakdown [11,12,29,30], and the scarce evidence from direct measurements of muscle protein breakdown (MPB) have reported conflicting results [12,30]. Particularly in overweight populations, it has been stated that the loss of muscle mass during a negative energy balance is caused more by a reduction in protein synthesis rather than an increase in protein degradation [13]. The high energy demand during severe energy deficits elicited by the combination of prolonged exercise and caloric restriction could evoke a greater activation of skeletal muscle protein degradation, but experimental data on humans are not available.

Skeletal muscle ALS and UPS signaling pathways could be upregulated after ultra-distance races, although results are inconclusive regarding the key signaling markers and the role played by the E3s [31,32]. However, it remains unknown how these signaling pathways are regulated in muscles of the upper extremities in humans, i.e., not involved in the exercise.

In humans, the mechanisms regulating protein degradation are influenced by feeding, and essential amino acids (EAA), leucine in particular, and have been shown to reduce skeletal muscle MuRF1 and MAFbx mRNA expression following acute endurance exercise [33] and resistance exercise [34]. Moreover, insulin and amino acids downregulate autophagosome formation in human skeletal muscle [35,36]. Nevertheless, it remains unknown how different levels of contractile activity and provision of amino acids modulate the UPS and ALS responses during severe energy deficits and the role of both systems on the loss of muscle mass during severe energy deficits.

**Figure 1:**
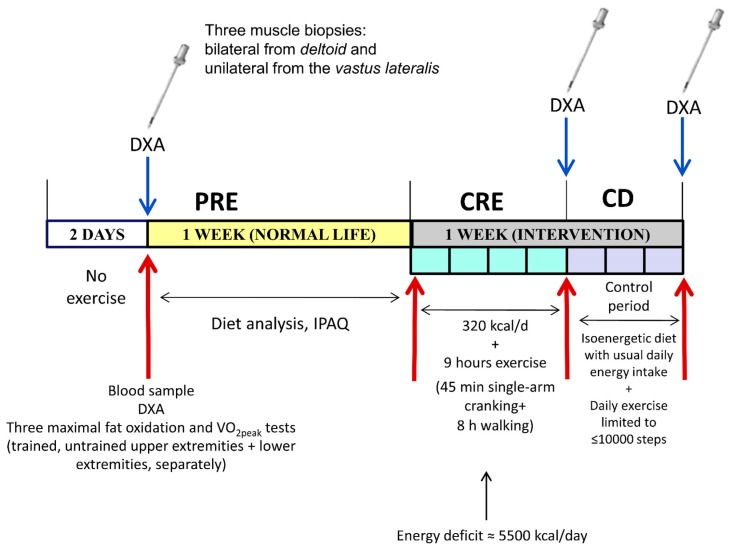
Schematic representation of the three phases of the intervention. PRE, before the intervention; CRE, four days of concomitant caloric restriction (3.2 kcal/day) and exercise (45 min of single-arm cranking and 8 h of walking each day); CD, three days on a diet isoenergetic to PRE phase with exercise limited to <10,000 steps per day; DXA, dual-energy X-ray absorptiometry; and IPAQ, International Physical Activity Questionnaire.

Therefore, the current investigation was designed to characterize the mechanisms by which exercise attenuates the loss of muscle mass during a severe energy deficit of ~5500 kcal/d for four days (Figure 1). The effect of local contractile activity on UPS and ALS signaling pathways was examined employing an intra-subject experimental design in which biopsies were taken from three different muscles subjected to different exercise volumes. Our subjects ingested a very low-calorie diet containing exclusively whey protein or sucrose, while performing 45 min of single-arm cranking (moderate volume) and eight hours of walking daily (high volume), with muscles in the contralateral arm serving as non-exercised controls. We hypothesized that exercise would attenuate the expression of markers of UPS and ALS during severe energy deficit in a dose-dependent fashion. Secondarily, we also hypothesized that compared to carbohydrates, the ingestion of whey protein alone would inhibit to a greater extent the proteolytic response.

## 2. Materials and Methods

A thorough characterization of our study population and experimental procedures can be found in the five previous published articles derived from the experiments carried out in this investigation [37,38,39,40,41]. A summary of our participants’ background characteristics is shown in Table 1. All participants received full oral and written information on the potential risks of taking part in the study and gave written informed consent prior to the start. The study was approved by the Regional Ethical Review Board of Umeå University (Umeå, Sweden).

### 2.1. Experimental Protocol

The protocol comprised the following three different experimental phases: A baseline phase (PRE), a phase of caloric restriction combined with exercise for four days (CRE), and a phase of controlled diet with reduced levels of exercise (CD) (Figure 1). The sample size was calculated to disclose any significant difference ≥1.5-fold larger than the coefficient of variation (which was <10% in most cases) between the mean values for any individual variable, with a significance level of *p* < 0.05 and statistical power of 0.8. Assessment of body composition by dual-energy X-ray absorptiometry (Lunar iDXA, GE Healthcare, Madison, WI, USA), extraction of 20 mL blood samples (in the supine position) and three muscle biopsies (one from each deltoid muscle, posterior portion, and one from the middle portion of the vastus lateralis) were obtained following a 12 h overnight fast during PRE. The biopsies following the CRE and CD phases were taken in the morning (i.e., 08:00 a.m.) on the next day after the end of the corresponding phase following a 12 h overnight fast (Figure 1).

Participants were randomly assigned to ingest a very low-calorie diet (0.8 g/kg body weight/day) consisting solely of sucrose (*n* = 7) or whey protein (*n* = 8) (Syntrax Nectar, Syntrax Innovations, Scott City, MO, USA) during caloric restriction phase (CRE). On each CRE day, participants performed 45 min of one-arm cranking (at 15% of maximal intensity), followed by eight hours of walking. The deltoid muscles were chosen as representative of upper limb musculature because their fiber type composition is similar to that of vastus lateralis [42], and both muscles adapt similarly to prolonged low-intensity endurance training [43]. It has also been reported that, despite a substantially higher proportion of type II fibers in the triceps brachii as compared to vastus lateralis, both muscles adapt to endurance training in a similar manner [44]. The whey protein solution also contained Na^+^ (308 mg/L) and K^+^ (370 mg/L), as did the sucrose solution (160 and 100 mg/L, respectively). Either solution was dissolved in 1.5 L containing minerals and split in three intakes of 0.5 L in the morning (just preceding arm-cranking), and, subsequently, at midday and 8 PM (at the end of the walk). Throughout the walks, groups were allowed to drink a hypotonic rehydrating solution containing Na^+^ (160 mg/L), Cl^−^ (200 mg/L), K^+^ (100 mg/L), citrate (700 mg/L), and sucrose (3g/L) *ad libitum*.

During the three-day CD phase, all participants daily ate three standardized meals reproducing their regular energy intake (as assessed by weighing all food ingested during the 7-day pre-test period), and their physical activity was restricted to a maximum of 10,000 steps. This phase was designed to allow replenishment of body water and stabilization of body weight.

### 2.2. Assessment of Physical Activity, Nutrition, and Body Composition

Daily energy expenditure due to physical activity was analyzed using the short version of the International Physical Activity Questionnaire [45]. During the 7-day PRE phase, the participants kept a dietary record, and their food intake was analyzed (Dietist XP, Kost & Näringsdata, Bromma, Sweden). During the CD phase, all participants were provided a diet with the same energy content as that recorded during PRE, and the food ingested was weighed. Energy intake was also calculated employing the Dietist XP program. During the PRE phase, the participants ingested a mixed diet (18.5%, 41.6%, and 39.9% protein, fat, and carbohydrate, respectively, in the sucrose group; and 19.5%, 38.3%, and 42.2%, protein, fat and carbohydrate, respectively, in the whey protein group) for a total energy intake of 2256 ± 513 and 2086 ± 450 kcal/d (means ± SD), for the sucrose and whey protein groups, respectively.

The energy deficit was calculated from the reduction in the energy content of the body using the changes in body composition, assigning energy values of 9416.8 and 884.3 kcal/kg to fat and lean mass, respectively [40,46,47].

### 2.3. Hormonal and Biochemical Analyses

After a 12 h overnight fast, 20 mL blood samples were drawn from an antecubital vein directly into Vacutainer tubes (REF: 368499; 368498; BD Vacutainer, Stockholm, Sweden). Some samples were collected in tubes containing EDTA and centrifuged for 5 min at 2000 *g* and 4 °C, to obtain plasma; while others were centrifuged for 10 min at 2000 *g* and 4 °C to prepare serum. All of these samples were aliquoted on tubes precooled on ice water and rapidly stored at −80 °C until analyzed.

The concentrations in serum of glucose, insulin, leptin, cortisol, total testosterone, free testosterone, and plasma amino acids were determined as previously reported [38,41]. HOMA index was calculated as the fasting plasma concentration of insulin (μU/mL) × the corresponding concentration of glucose (mmol/L)/22.5.

### 2.4. Biopsy Sampling

Three muscle biopsies were taken from the middle portion of each deltoid muscle and vastus lateralis using Bergstrom’s technique with suction, as described elsewhere [37]. After disinfection of the skin, 1 mL to 2 mL local anesthetic (Lidocaine 2%) was injected into the skin and subcutaneous tissue, taking care not to penetrate below the superficial fascia. After that, a 6 mm to 7 mm incision was made, and the biopsy Bergstrom-type needle inserted. The muscle sample (~100 mg) was dissected free of any debris and fat tissue present and immediately frozen in liquid nitrogen and stored at −80 °C until further analysis.

### 2.5. Protein Extraction and Western Blotting

Extracts of muscle protein were prepared as previously described [48], and total protein content quantified using the bicinchoninic acid assay [49]. Briefly, 30 mg of muscle was homogenized in urea lysis buffer (6 M urea, 1% SDS and 1X cOmplete protease inhibitor and phosphatases PhosphoStop 1X) and the lysate then centrifuged for 12 min at 25,200 *g* at 16 °C. The resulting supernatant containing the protein fraction, was diluted with electrophoresis loading buffer (62.50 mM Tris-HCl, pH 6.8, 2.3% SDS, 10% glycerol, 5% β-mercaptoethanol, and bromophenol blue).

The optimal antibody concentration and the total protein amount to be loaded was first determined by loading a gradient of protein extracts at concentrations between 15 and 35 μg. The linear relation between total protein concentration loaded and quantitative band intensity was calculated. After confirming linearity in this range, equal amounts for the same protein determination (30 to 35 μg) of each sample were electrophoresed with SDS-PAGE and transferred to Immun-Blot PVDF membranes for protein blotting (Bio-Rad Laboratories, Hercules, CA, USA). To compensate for variability between gels, the nine samples from each subject and a control sample in quadruplicate (human muscle) were equally loaded onto the same gel. When the coefficient of variation for the control samples was >20% the blot was repeated. The sample protein bands were normalized to the mean value of the control sample.

Membranes were blocked for one hour in 4% bovine serum albumin in Tris-buffered saline containing 0.1% Tween 20 (TBS-T) (BSA-blocking buffer) and incubated overnight at 4 °C with primary antibodies. In the case of the protein markers whose phosphorylation was assessed, the membranes were initially incubated with the phospho-specific antibodies and, after detection, the antibodies were stripped off using a buffer containing Tris-HCL 1 M pH 6.7, SDS 20%, β-mercaptoethanol 14.3 M and H_2_O and subsequently re-blocked and re-incubated with antibodies against the total protein. Antibodies were diluted in 4% BSA-blocking buffer or 5% Blotto blocking buffer. After incubation with primary antibodies, the membranes were incubated with an HRP-conjugated antibody (diluted 1:5000 in Blotto blocking buffer in all instances) and subsequent chemiluminescent visualization with Clarity™ Western ECL Substrate (Bio-Rad Laboratories (Hemel Hempstead, Hertfordshire, UK) using the ChemiDoc Touch Imaging System (Bio-Rad Laboratories, Hercules, CA, USA) until saturation of the signal. Finally, band densitometric data were quantified in the exposition immediately prior to saturation of the signal with Image Lab© software (Bio-Rad Laboratories, Hercules, CA, USA). To control for differences in loading and transfer efficiency, membranes were stained with Reactive Brown and quantified. In order to verify equal loading, the blots were stripped and probed with a monoclonal mouse anti-α-tubulin antibody. No significant changes were observed in Reactive Brown total protein amount nor in α-tubulin protein levels (data not shown).

### 2.6. Materials

The complete protease inhibitor cocktail and PhosSTOP phosphatases inhibitor cocktail were obtained from Roche Diagnostics (Mannheim, Germany; #04693116001 and #04906845001, respectively). The Immun-Blot PVDF membranes, the Immun-StarTM Western CTM and the Protein Plus Precision All Blue Standards were from Bio-Rad Laboratories (Hemel Hempstead Hertfordshire, UK). The corresponding catalog numbers of primary antibodies were as follows: anti-phospho-AMPKα (Ser172), no. 2531, anti-phospho-Beclin 1 (Ser15), no.8466, anti-phospho-FoxO1 (Ser256), no. 9461, anti-phospho-FoxO3 (Ser253), no. 9466, anti-AMPKα, no. 2532; and anti-FoxO1, no. 9454, were purchased from Cell Signaling Technology (Danvers, MA, USA). Anti-eIF3f, no. ab74568; Anti-LC3B, no. ab48394; anti-MuRF1, no. ab77577; anti-p62/SQSTM1, no. ab56416; and anti-phospho-ULK1 (Ser555), no. ab229537 were purchased from Abcam (Cambridge, UK). Anti-MAFbx, no. sc166806 and anti- FKHRL1, no. sc11351 were purchased from Santa Cruz Biotechnology (Dallas, TX, USA), and anti-Atg1/ULK1, no. A7481 and anti-α-tubulin, no. T5168 was obtained from Sigma-Aldrich (St. Louis, MO, USA). The secondary HRP-conjugated goat anti-rabbit (no. sc2030 and sc2004), chicken anti-rabbit (sc516087) and goat anti-mouse (sc2031) antibodies were from Santa Cruz Biotechnology, and the goat anti-rabbit (no. 111-035-144) was purchased from Jackson ImmunoResearch (West Grove, PA, USA).

### 2.7. Statistical Analysis

The normality of data was checked using the Shapiro–Wilks tests, and when necessary, data were transformed logarithmically before analysis. A three-way repeated measures ANOVA with time (PRE, CRE, and CD) and muscle (control deltoid, trained deltoid, and vastus lateralis) as within-subject factors, and the two different diets (sucrose versus whey protein) as a between-subjects factor was performed as the primary statistical test. The same type of three-way repeated measures ANOVAs were also conducted with two levels for muscle to distinguish between upper and lower extremity muscle adaptations (the average of both deltoid muscles vs. vastus lateralis). Likewise, to compare the responses of the exercised and non-exercise muscles, the same type of three-way repeated measures ANOVAs was carried out with two levels for the muscle factor defined as exercised vs. non-exercised muscles (i.e., the non-exercised deltoid vs. the average of the exercised deltoid and vastus lateralis). To study the overall signaling response in all muscles, the average of the three biopsies was calculated, and a two-way repeated measures ANOVA was run with time (PRE, CRE, and CD) as a within-subject factor and the two different diets (sucrose versus whey protein) as the between-subjects factor. The Mauchly’s test of sphericity was run before the ANOVA. In the case of violation of the sphericity assumption, the degrees of freedom were adjusted according to the Huynh and Feldt test. When a significant main effect or interaction was observed, pairwise comparisons at specific time points were adjusted for multiple comparisons using the Holm–Bonferroni procedure.

Pearson’s correlation analysis was used to examine the associations between changes in signaling and hormones or amino acids using all subjects (i.e., without a separate analysis for the sucrose and whey protein groups). For this purpose, a mean signaling response was calculated for each subject (mean of the three biopsies) and the study phase. The changes from PRE to CRE (*n* = 15) and PRE to CD (*n* = 15) were calculated, and these changes were tested for association with the corresponding changes in hormones or amino acids. Additionally, the same approach was used for the average of both deltoid muscles, to represent the mean changes in signaling of the arms. The changes in signaling in the vastus lateralis were also tested independently since this muscle was submitted to an extremely high volume of exercise. Additional correlations were performed combining the changes from PRE to CRE, and from PRE to CD (*n* = 30).

Results are expressed as mean ± standard deviations (SD) unless otherwise stated. Statistical significance was set at *p* < 0.05. All statistical analyses were performed using SPSS v.15.0 for Windows (SPSS Inc., Chicago, IL, USA).

## 3. Results

### 3.1. Exercise Preserved Muscle Mass in a Dose-Dependent Fashion without Significant Changes in Protein Synthesis Signaling

The responses regarding body composition, hormonal and metabolic status, and performance have been previously described in detail [37,40,41] and will only be summarized here. Following CRE, lean mass was reduced by 6% and 4% in the upper extremities and the lower extremities, respectively (both *p* < 0.001 and *p* < 0.05 for time × extremity interaction), with no significant differences between the two diets (*p* = 0.34). Despite a mild negative energy balance during control diet (CD) phase, ~50% of the muscle mass lost from the four extremities during the caloric restriction and exercise phase (CRE) was recovered by replenishment of body water during CD [38,40]. Accordingly, following the three days of control diet and limited exercise, the relative losses in lean mass of the lower extremities and trained upper extremities were 57% and 29% less, respectively, than observed in the untrained upper extremities (*p* < 0.05) [41].

No significant changes were found in the Akt/mTor/p70S6K signaling pathway regulating protein synthesis following the four-day intervention in any of the muscles studied, regardless of the ingestion of whey protein [38]. The vastus lateralis showed a larger percentage of myosin heavy chain (MHC) isoforms than the deltoid (52.2% ± 14.4% and 41.5% ± 5%, respectively, *p* < 0.01) [37]. The proportion of MHC were not modified by the intervention [37].

### 3.2. Exercise Attenuated the Increase of Ser256 FoxO1 and Ser253 FoxO3a Elicited by the Negative Energy Balance, but Had No Significant Effect on the Levels of MuRF1, MAFbx, and Eif3f Protein in Skeletal Muscle, Regardless of Protein Supplementation

**Figure 2:**
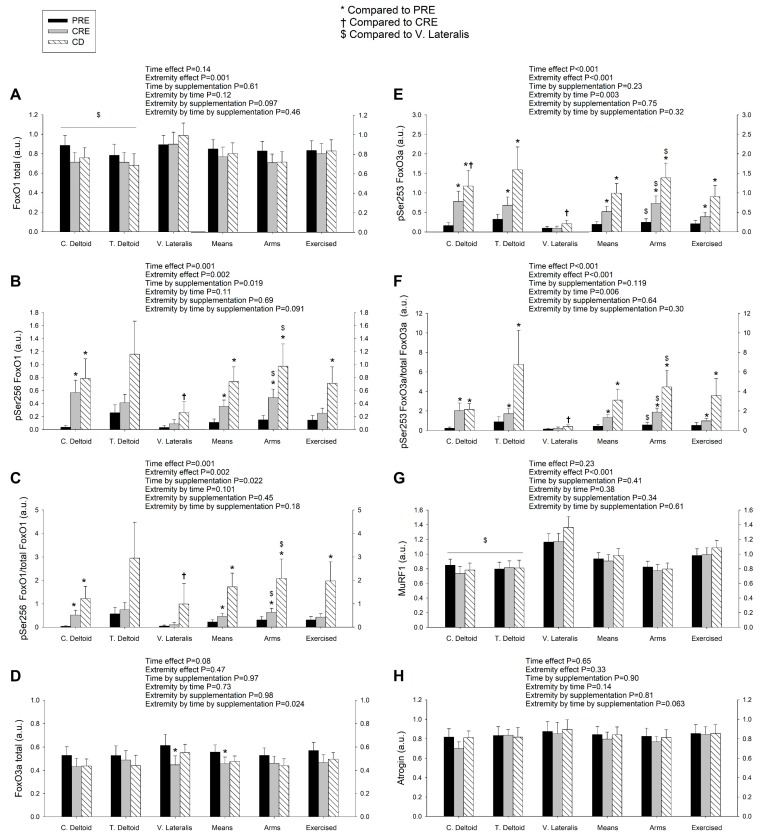
Exercise attenuates the increase of Ser256 FoxO1 and Ser253 FoxO3a elicited by severe energy deficit, without altering the markers of degradation by the ubiquitin-proteasome system. Protein expression levels of total, phosphorylated, and fractional phosphorylation of FoxO1 (**A**–**C**) and FoxO3a (**D**–**F**), E3 ligases MuRF1 (**G**) and MAFbx (**H**) in skeletal muscle following the three experimental phases. The various phases of the intervention are explained in Figure 1. PRE, before the intervention; CRE, caloric restriction and exercise; CD, control diet; C. Deltoid, control deltoid; T. Deltoid, trained deltoid; V. Lateralis, vastus lateralis; Means, (C. Deltoid + T. Deltoid + vastus lateralis)/3; Deltoid muscles, (C. Deltoid + T. Deltoid)/2; and Exercised, (T. Deltoid + vastus lateralis)/2. *n* = 7 and *n* = 8 for the sucrose and whey protein groups, respectively. The statistical analysis was performed with logarithmically transformed data for all proteins except FoxO1 and MuRF1. The values shown are means ± standard errors and expressed in arbitrary units (a.u.). *, *p* < 0.05 compared to PRE; ^†^, *p* < 0.05 compared to CRE; and ^$^, *p* < 0.05 compared to vastus lateralis. Note: The amount of Ser256 FoxO1 was almost undetectable in the vastus lateralis of the representative subject.

FoxO1 expression was larger in the vastus lateralis than the deltoid muscles (Figure 2A). Although there were non-significant changes in total expression levels of FoxO1 after CRE and CD, the mean expression in the deltoid muscles (mean of both arms) tended to decline while the vastus lateralis changed in the opposite direction (extremity by time interaction, *p* = 0.026) (Figure 2A). The changes in the expression of FoxO1 in the deltoid muscles (mean of both arms) from before the intervention (PRE) to CRE were positively associated with the corresponding increase of the cortisol/total testosterone ratio (*r* = 0.54, *p* = 0.04, and *n* = 15) and negatively with the changes in total testosterone (*r* = −0.63, *p* = 0.012, and *n* = 15), but no association was observed with cortisol alone (*r* = −0.03, *p* = 0.9, and *n* = 15) (Figure S1A and S1B). FoxO1 phosphorylation at Ser256 increased in the deltoid muscles following CRE and CD, and the vastus lateralis following CD (Figure 2B). No significant correlations were observed between changes in Ser256 FoxO1 in the deltoid muscles and those of hormones and amino acids. In the vastus lateralis, the change in Ser256 FoxO1 expression from PRE to CRE was positively associated with the corresponding change in cortisol (*r* = 0.55, *p* = 0.034, and *n* = 15), cortisol/total testosterone (*r* = 0.77, *p* = 0.001, and *n* = 15), cortisol/free testosterone (*r* = 0.84, *p* < 0.001, and *n* = 15), insulin (*r* = 0.60, *p* = 0.017, and *n* = 15) and leucine (*r* = 0.60, *p* = 0.018, and *n* = 15) suggesting that cortisol, the catabolic index, insulin, and leucine inhibited FoxO1 signaling in the vastus lateralis (Figure S1C–S1F). The fractional phosphorylation of FoxO1 was markedly elevated in the deltoid muscles and blunted in the vastus lateralis after CRE and CD (Figure 2C). The inhibitory phosphorylation of FoxO1 was greater after the ingestion of proteins (time by supplementation, *p* = 0.022). These results indicate that a high volume of exercise counteracts the inhibition of FoxO1 signaling in skeletal muscle elicited by a severe energy deficit, whereas the ingestion of proteins counteracts this effect partly.

Similar to FoxO1, FoxO3a total protein was reduced after CRE, primarily due to a lower expression in the vastus lateralis of the subjects ingesting protein (extremity by time by supplementation interaction, *p* = 0.024) (Figure 2D). The Ser253 FoxO3a and the ratio Ser253 FoxO3a/total FoxO3a rose after CRE and increased further following CD, with a greater response in the deltoid muscles than in the vastus lateralis (extremity by time interaction, both *p* < 0.01) without significant differences between groups (Figure 2E,F). Altogether, these findings are compatible with an increase in FoxO3a inhibition with the energy deficit that was attenuated by a high exercise volume, while the ingestion of proteins counteracted this effect partly.

**Figure 3:**
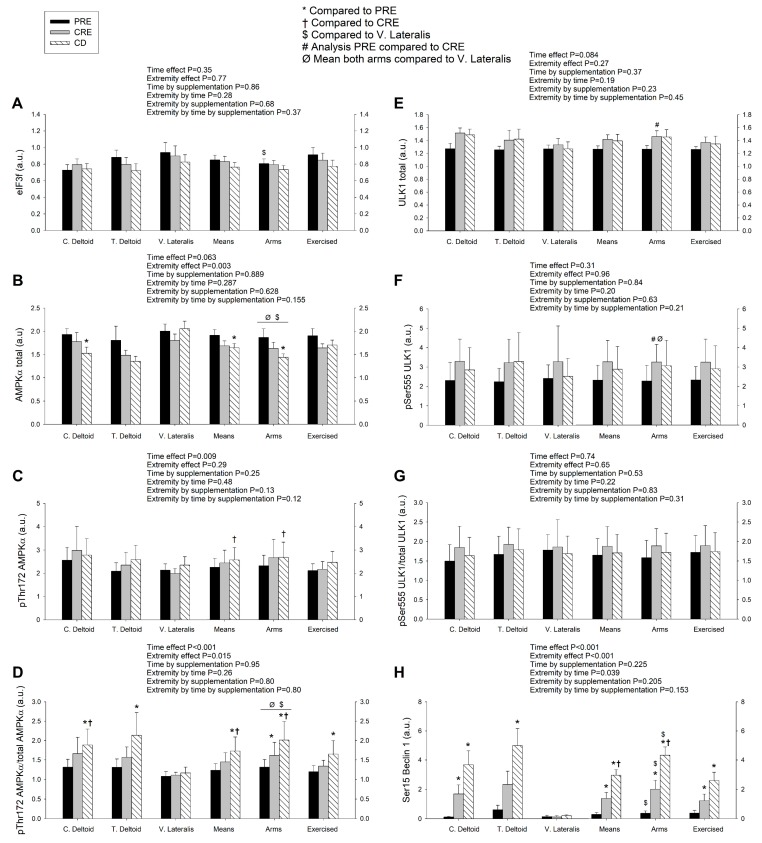
A high volume of exercise prevents the phosphorylation Thr172 AMPK, Ser555 ULK1, and Ser15 Beclin 1 in skeletal muscle, blunting the autophagic response and preserving lean mass during a severe energy deficit. Protein expression levels of eIF3f (**A**); total and phosphorylated AMPKα (**B**–**D**), and ULK1 (**E**–**G**) and the corresponding fractional phosphorylation ratios, and phosphorylated Beclin 1 (**H**) in skeletal muscle following the three experimental phases. The various phases of the intervention are explained in Figure 1. PRE, before the intervention; CRE, caloric restriction and exercise; CD, control diet; C. Deltoid, control deltoid; T. Deltoid, trained deltoid; V. Lateralis, vastus lateralis; Means, (C. Deltoid + T. Deltoid + vastus lateralis)/3; Deltoid muscles, (C. Deltoid + T. Deltoid)/2; Exercised, (T. Deltoid + vastus lateralis)/2. *n* = 7 and *n* = 8 for the sucrose and whey protein groups, respectively. The statistical analysis was performed with logarithmically transformed data for all proteins. The values shown are means ± standard errors and expressed in arbitrary units (a.u.). *, *p* < 0.05 compared to PRE; ^†^, *p* < 0.05 compared to CRE; ^$^, *p* < 0.05 compared to vastus lateralis; ^#^, *p* < 0.05 PRE compared to CRE (only PRE and CRE conditions included in the ANOVA); and ^Ø^, *p* < 0.05 mean both deltoid compared to vastus lateralis.

Despite this reduced FoxO1/3a signaling, the ubiquitin E3-ligases MuRF1 and MAFbx protein expression remained unchanged in all muscles (Figure 2G,H). The deltoid muscles had lower MuRF1 expression levels than the vastus lateralis (extremity effect, *p* < 0.001). We also examined the protein levels of Eif3f, which is ubiquitinated and targeted for proteasomal degradation by MAFbx. As expected, Eif3f remained unaltered after CRE and CD in all muscles (Figure 3A).

### 3.3. A High Volume of Exercise Prevents the Phosphorylation of Thr172 AMPK, Ser555 ULK1, and Ser15 Beclin 1 in Skeletal Muscle, Blunting the Autophagic Response and Preserving Lean Mass during a Severe Energy Deficit

Total AMPKα protein expression was larger in the vastus lateralis than the deltoid muscles, and was reduced from PRE to CD, regardless of the supplement ingested (Figure 3B). Since this response was similar in both deltoid muscles, the average of the two deltoid muscles was compared to the response observed in the vastus lateralis. From PRE to CRE, total AMPK tended to decrease in the deltoid muscles (*p* = 0.067) and it was significantly reduced, from PRE to CD (*p* = 0.028). No significant changes in total AMPK were observed in the vastus lateralis (mean of both arms by vastus lateralis by time interaction, *p* = 0.05). Overall, there was an increase in the amount of the phosphorylated form of AMPKα at Thr172 over time (*p* = 0.009) (Figure 3C). The ratio pThr172 AMPKα/total AMPKα increased from PRE to CRE and from CRE to CD in the deltoid muscles (mean of both arms by vastus lateralis interaction, *p* = 0.07), while no such increase was observed in the vastus lateralis (Figure 3D), being the response observed not influenced by the ingestion of proteins.

AMPK promotes autophagy by directly phosphorylating and activating ULK1. Globally, no significant changes in total and phosphorylated at Ser555 ULK1 protein expression were observed (Figure 3E,F). A secondary analysis was carried out including only PRE and CRE values, to capture the effects exclusively due to the exercise phase with caloric restriction. From PRE to CRE, total ULK1 was increased in the deltoid muscles (time effect, *p* < 0.001) (Figure 3E). Compared to PRE, Ser555 ULK1 (mean of both deltoid) was increased in the deltoid muscles but not in the vastus lateralis after CRE (extremity by time interaction, *p* = 0.049) (Figure 3F). The ratio pSer555 ULK1/total ULK1 (mean of both deltoid) tended to increase (*p* = 0.10) while it remained unchanged in the vastus lateralis (extremity by time interaction, *p* = 0.046) (Figure 3G).

In agreement with greater activation of ULK1 in the deltoid muscles, Beclin 1 which is a substrate for ULK1, was markedly phosphorylated at Ser15 in the deltoid muscles after CRE and even further phosphorylated after CD (time effect, *p* < 0.001), while it remained unchanged in the vastus lateralis (extremity by time interaction, *p* = 0.039). The type of supplementation had no apparent influence on Beclin 1 phosphorylation changes (time by supplementation interaction, *p* = 0.23) (Figure 3H).

### 3.4. LC3BI Expression in Skeletal Muscle Increases with a Severe Energy Deficit, without Significant Changes in LC3BII nor the LC3BII/LCBI Ratio, Regardless of the Ingestion of Proteins

**Figure 4:**
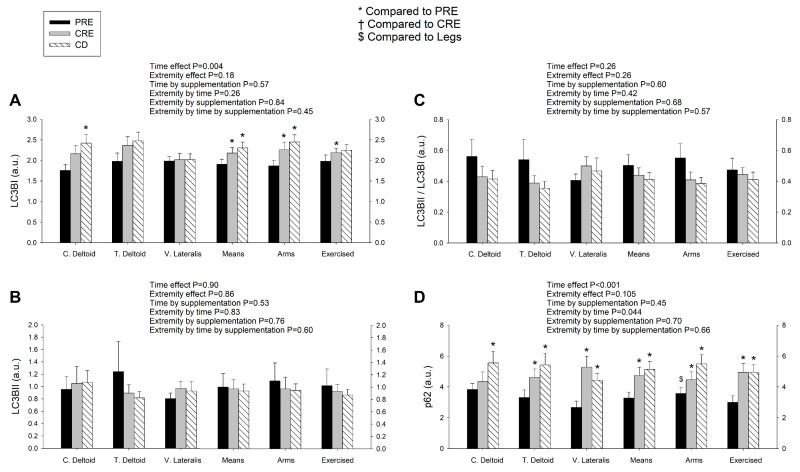
LC3BI expression in skeletal muscle increases with a severe energy deficit, but LC3BII and the LC3BII/LCBI ratio remain unaltered, while the augmented levels of p62/SQSTM1 are amplified by exercise in a dose-dependent fashion. Protein expression levels of of LC3BI (**A**), LC3BII (**B**), ratio LC3BII/LCBI (**C**), and p62/SQSTM1 (**D**) in skeletal muscle following the three experimental phases. The various phases of the intervention are explained in Figure 1. PRE, before the intervention; CRE, caloric restriction and exercise; CD, control diet; C. Deltoid, control deltoid; T. Deltoid, trained deltoid; V. Lateralis, vastus lateralis; Means, (C. Deltoid + T. Deltoid + vastus lateralis)/3; Deltoid muscles, (C. Deltoid + T. Deltoid)/2; Exercised, (T. Deltoid + vastus lateralis)/2. *n* = 7 and *n* = 8 for the sucrose and whey protein groups, respectively. The statistical analysis was performed with logarithmically transformed data for all proteins. The values shown are means ± standard errors and expressed in arbitrary units (a.u.). *, *p* < 0.05 compared to PRE; and ^$^, *p* < 0.05 compared to vastus lateralis.

The autophagosome-bound LC3BII levels and the ratio LC3BII/I can be used as markers of autophagosome number and autophagic flux [28], respectively. Compared to the PRE, the mean levels of LC3BI were significantly increased after CRE and CD (time effect, *p* = 0.004), with this effect being likely due to the increase in the untrained and trained deltoid muscles following CRE and CD, with similar responses in both supplementation groups (Figure 4A) (extremity by time interaction *p* = 0.09 and ANOVA for repeated measures done with the mean of both deltoid for this analysis). However, neither the protein levels of LC3BII nor the ratio LC3BII/LCBI were significantly modified by the intervention in either group (Figure 4B,C).

#### 3.5. p62/SQSTM1 Expression Increases with a Negative Energy Balance and this Response Is Amplified by Exercise in a Dose-Dependent Fashion

**Figure 5:**
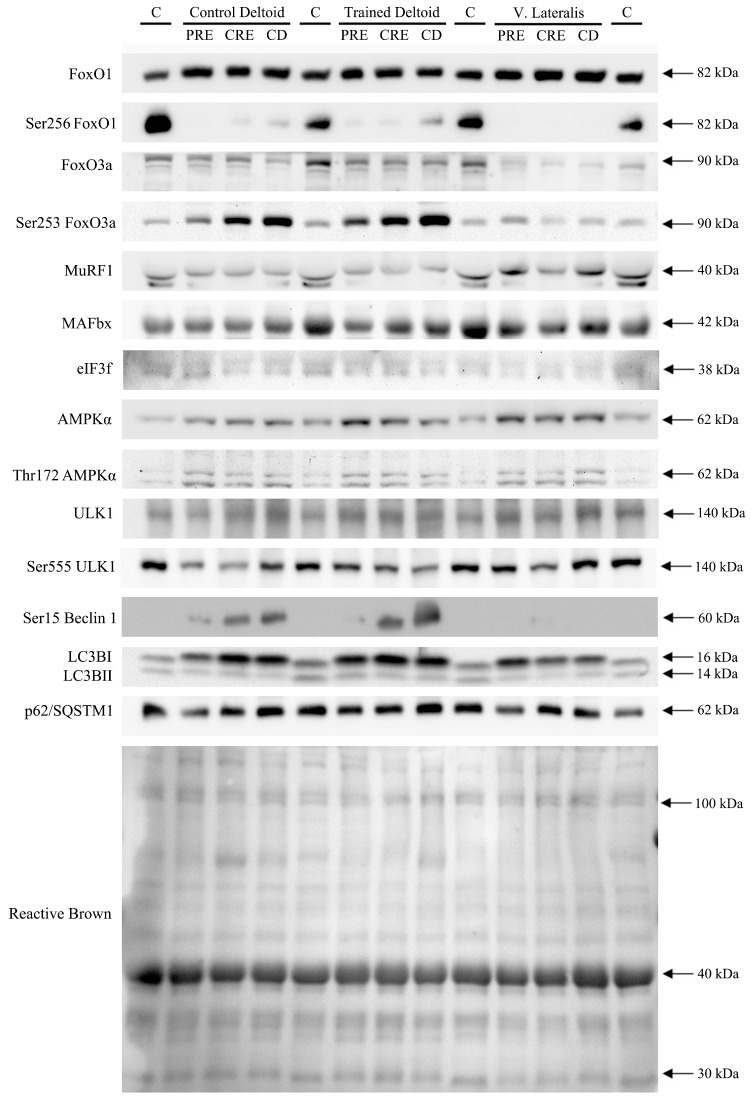
Representative immunoblot images of all proteins studied and their regulatory phosphorylations and the total amount of protein loaded (Reactive Brown staining) from a single participant in the study belonging to the whey protein group. From top to bottom: FoxO1, pSer256 FoxO1, FoxO3, pSer253 FoxO3, MuRF1, MAFbx, eIF3f, AMPKα, pThr172 AMPKα, ULK1, pSer555 ULK1, pSer15 Beclin 1, LC3BI, LC3BII, p62/SQSTM1 and Reactive Brown (as protein loading control). Quadruplicate assays of a non-intervention human sample were included on each gel as a loading control. The various phases of the intervention are explained in Figure 1. PRE, before the intervention; CRE, caloric restriction and exercise; CD, control diet. Estimated molecular weights are indicated by arrows.

In the final step of autophagy, p62 (also called SQSTM1), an acceptor for ubiquitinated substrates, undergoes autolysosomal degradation [50]. A reduction of p62/SQSTM1 is suggestive of increased autophagic flux while the accumulation of p62/SQSTM1 is considered a marker of autophagy inhibition [51]. At PRE, the p62 expression levels were higher in the deltoid muscles than the vastus lateralis (Figure 4D). Protein expression of p62 was increased after CRE and CD, with this response being larger for the vastus lateralis than the deltoid muscles (extremity by time interaction, *p* = 0.044), which is compatible with inhibition of autophagic flux in the vastus lateralis. These responses were similar for the sucrose and whey protein groups. Representative immunoblots are presented in Figure 5.

### 3.6. The Autophagic Response to a Severe Energy Deficit Is Positively Associated with the Increase in the Catabolic Index (Cortisol/Testosterone), Negatively Associated with Insulin Changes, and Partly Modulated by Circulating Essential Amino Acids

The changes in the mean expression (mean of the three muscles) of Ser555 ULK1/total ULK1, from PRE to CRE and from PRE to CD (*n* = 30), were linearly related to the corresponding changes in insulin (*r* = −0.41, *p* = 0.025, and *n* = 30) and homeostasis model assessment (HOMA) (*r* = −0.39, *p* = 0.032, and *n* = 30) (Figure S2A and S2B). The changes in the mean expression (average of the three muscles) of Ser555 ULK1/total ULK1, from PRE to CRE, were associated with the corresponding changes in the circulating levels of branched-chain amino acids (BCAA) (*r* = −0.54, *p* = 0.037, and *n* = 15) and leucine (*r* = −0.51, *p* = 0.052, and *n* = 15) (Figure S2C and S2D). This suggests an overall inhibitory action of insulin on the induction of autophagy in all muscles. To find out whether the amount of exercise might have influenced the responsiveness of individual skeletal muscles to endocrine signals and circulating amino acids, we also studied each extremity separately. In the vastus lateralis, the changes in the Ser555 ULK1/total ULK1 ratio, from PRE to CRE and from PRE to CD, were associated with the corresponding changes in circulating BCAA (*r* = −0.60, *p* = 0.019, and *n* = 15; and *r* = −0.72, *p* = 0.003, and *n* = 15, respectively), EAA (*r* = −0.53, *p* = 0.041, and *n* = 15; and *r* = −0.80, and *p* < 0.001, *n* = 15, respectively), leucine (*r* = −0.65, *p* = 0.009, and *n* = 15; and *r* = −0.36, *p* = 0.185 and *n* = 15, respectively), and insulin (*r* = −0.34, *p* = 0.207, and *n* = 15; and *r* = −0.67, *p* = 0.006, and *n* = 15) (Figure S2E–S2G). No significant correlations were observed between the changes in Ser555 ULK1/total ULK1 ratio in the deltoid muscles and the changes in hormones or amino acids. Overall these associations are compatible with an inhibitory action of insulin and amino acids, which might have been facilitated by the large amount of exercise performed by the lower extremities. A potentially inhibitory influence of the anabolic hormones insulin and testosterone on autophagy was also observed at other levels of the autophagy signaling cascade. For example, the reductions in the expression of FoxO3a (mean of the three muscles) from PRE to CRE were associated with the corresponding changes in insulin (*r* = −0.66, *p* = 0.007, and *n* = 15) and HOMA (*r* = −0.70, *p* = 0.004, and *n* = 15) (Figure S1G and S1H). Likewise, the final steps of the autophagy signaling pathway seem to be also downregulated by insulin, as suggested by the association observed between the changes in insulin and the changes in the deltoid muscles’ expression (mean of both deltoids) of the ratio LC3BII/LCBI, from PRE to CRE and from PRE to CD (*r* = −0.45, *p* = 0.012, and *n* = 30) (Figure S2H).

## 4. Discussion

**Figure 6:**
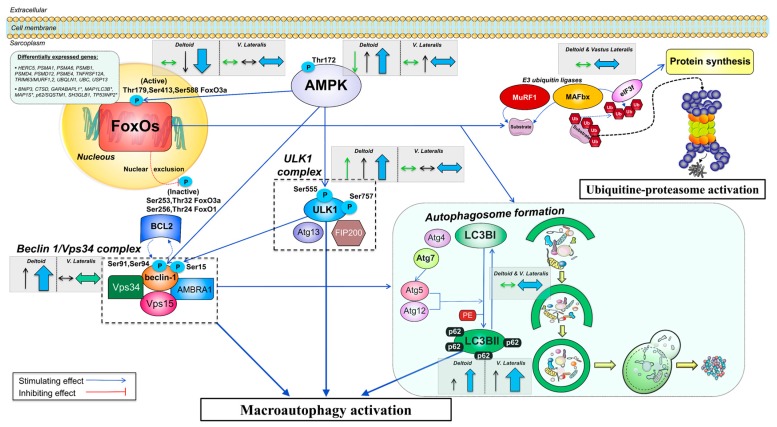
Schematic representation of the regulation of the autophagy-lysosome system in response to severe energy deficit and its modulation by exercise. An upregulation of numerous genes involved in the ubiquitin-proteasome and the autophagy-lysosome degradation pathways is seen after the CRE phase, primarily in the vastus lateralis. Elevated levels of cortisol with low insulin, testosterone, and leptin levels did not prevent increased inhibitory phosphorylation of FoxO1 and FoxO3a in the deltoid muscles, which was blunted by prolonged low-intensity exercise in the vastus lateralis, despite inactivation of the PI3K/Akt/mTOR pathway [52]. FoxOs orchestrate the activation of the ubiquitin-proteasome and autophagy-lysosomal degradation pathways. Upon FoxOs unchanged or reduced activation, the levels of E3 ligases MuRF1 and MAFbx are not augmented, limiting the ubiquitin-mediated tagging of proteins for proteasomal degradation. The central role in the antagonistic regulation of catabolic and anabolic signals driven by eIF3f is not altered as a consequence of its MAFbx-dependent degradation. AMPK can activate FoxO3a via phosphorylation or independently stimulate degradation via the autophagy-lysosomal pathway. A severe energy deficit induces autophagy activation via higher Thr172 AMPKα in all muscles, although the augmented fractional phosphorylation at Thr172 solely present in the deltoid muscles indicates inactivation by a large exercise volume in the lower extremities. AMPKα directly increases activation of ULK1 through phosphorylation at Ser555, an effect abrogated in the vastus lateralis by high-volume exercise. AMPK and ULK1 propagate downstream signaling by altering the regulation of the Beclin 1/Vps34 complex, whose fine integration and functioning is required for complete autophagosome formation. An increased phosphorylated Beclin 1 at Ser15 can be sufficient to regulate the levels of the entire complex, and likely the augmented phosphorylation present only in the deltoid muscles elicits stimulation of autophagy induction, which was not present in the vastus lateralis. No sign of increased LC3BII or conversion of LC3BI to LC3BII is suggestive of an overall unchanged rate of autophagosome formation. The enhanced accumulation of the sequestosome 1 (p62/SQSTM1) in all muscles but preferentially in the vastus lateralis, is supportive of an inhibition of autophagic flux modulated by the volume of exercise performed by the skeletal muscles, as p62/SQSTM1 is an acceptor of ubiquitinated substrates whose expression should be lowered following lysosomal degradation. The arrows included inside dashed grey boxes illustrate the overall protein expression changes in this investigation split in the deltoid muscles (mean of both arms) and vastus lateralis and are shown beside the specific markers. Thin arrows in green (total form) and black (phosphorylated form) depict the overall direction of the outcomes (increase/decrease) for the particular muscle group. Thick arrows in blue depict the overall effect on stimulation/inhibition of the ubiquitin-proteasome or autophagy-lysosomal degradation pathways. The size of the arrow is representative of the magnitude of the change. Activatory/inhibitory actions are represented by blue/red connecting lines (dashed if changes in location are present). Differentially expressed genes refer to an upregulation unless an asterisk (*) is placed on the gene name (downregulation). Abbreviations not defined in the text: AMBRA1, activating molecule in BECN1 regulated autophagy protein 1; Atg4/5/7/12/13, autophagy-related 4/5/7/12/13; BCL2, B-cell lymphoma 2; FIP200, focal adhesion kinase family-interacting protein of 200 kDa; Vps15, vacuole protein sortin 15.

This study was designed to determine the molecular mechanisms by which exercise attenuates the losses of muscle mass during a severe energy deficit in humans. For this purpose, we have examined the main proteolytic signaling pathways using an intrasubject design where three muscles were submitted to the same whole-body energy deficit but different amounts of exercise. The present investigation indicates that the main molecular catabolic pathway activated during a severe energy deficit (~5500 kcal/d for four days) is the autophagy-lysosome system, while the ubiquitin-proteasome pathway does not seem to be involved in our experimental conditions. Importantly, our study reveals that the main mechanism by which exercise preserves lean mass during a severe energy deficit is by blunting the activation of the autophagy-lysosome system, acting simultaneously on several of the signaling events necessary for complete autophagy. This interpretation is supported by a blunted phosphorylation of AMPK, ULK1, and Beclin 1, accompanied by an unaltered ratio LC3BII/LC3BI combined with a superior increase in the levels of p62 in the vastus lateralis (Figure 6). These molecular events are compatible with a reduction of the autophagy flux occurring primarily in the vastus lateralis, which was subjected to an extraordinary amount of exercise. Consequently, the lower extremities lost proportionally less muscle mass than the upper extremities. Although protein intake had some inhibitory actions on the activation of the autophagy signaling pathway, these effects were insufficient to impede the loss of muscle mass and did not add to the inhibitory action of exercise on autophagy during a severe energy deficit. This resulted in no clear advantage of the ingestion of proteins to preserve muscle mass, regardless of the amount of exercise done by each muscle.

### 4.1. The Activation of the Signaling Cascades Regulating Protein Degradation Is Primarily Driven By AMPK and Not FoxOs under a Severe Energy Deficit

AMPK potently stimulates autophagy by activating ULK1, impeding mTOR inactivation of ULK1, and promoting FoxO3a signaling through its phosphorylation at Ser588 [53]. AMPK phosphorylation increased with the intervention, but this effect was blunted in the vastus lateralis, and consequently, Ser555 ULK1 was also less phosphorylated in vastus lateralis than the deltoid muscles after the four-day severe energy deficit. This is supported by the unchanged levels of total and phosphorylated (Ser2448) mTOR after the four-day intervention [38]. In agreement, we have previously reported upregulation of autophagy-related gene expression in the vastus lateralis of our subjects, after CRE [39]. In brief, the most differentially expressed autophagy genes were BNIP3 (1.8-fold and Padj < 0.001), CTSD (1.8 fold and Padj < 0.001) and SQSTM1 (1.7-fold and Padj < 0.001) which is in agreement with previous findings reporting induction of autophagy-related genes in human skeletal muscle following long-lasting endurance protocols [54].

The current investigation has shown that FoxO1 and FoxO3a are inhibited in the deltoid muscles via phosphorylation following the four-day intervention, and further after CD. This inhibitory action was blunted for both FoxOs in the vastus lateralis following CRE, and largely attenuated as compared to the deltoid muscles after CD. Phosphorylation at Ser256 and Ser 253 of FoxO1 and FoxO3a, respectively, is carried out by Akt/protein kinase B (PKB) [30] and leads to nuclear exclusion and repression of the FoxOs-regulated transcriptional programs. Akt-mediated FoxOs inhibitory phosphorylations are stimulated by insulin, and accordingly positive associations were observed between the increase in FoxO1 phosphorylation in the vastus lateralis and the corresponding changes in circulating insulin. Although we reported a non-increased fractional pSer473Akt/total Akt after the two experimental phases [38], a transient activation of Akt before the biopsies could not be ruled out as there was a significant increase of total Akt in the deltoid muscles after CD, and downstream targets of the Akt/mTORC1 pathway were also significantly increased [38]. An alternative mechanism that could explain an increased level of FoxO3a and FoxO1 inhibitory phosphorylations is the activation of serum- and glucocorticoid-inducible kinase 1 (SGK1) [55], which could have been mediated by the increased concentration of cortisol observed during the energy deficit phase [40]. In agreement, the changes in vastus lateralis Ser256 FoxO1, from PRE to CRE, were strongly associated with the increase in circulating cortisol and even more closely with the rise in cortisol/free testosterone ratio. Altogether, the two main mechanisms regulating FoxOs inhibition seem to act concertedly to prevent FoxOs activation in skeletal muscle during a severe energy deficit, while a high volume of exercise attenuates this effect in the vastus lateralis. Potential inhibition of protein phosphatase 2A (PPA2), which dephosphorylates and activates FoxO3a in HeLa cells [56], is unlikely since FoxO1 phosphorylation was also increased and associated with the main regulatory hormonal mechanisms stimulating FoxOs phosphorylation.

The inhibition of FoxOs elicited by low-intensity exercise with severe energy deficit may have interesting clinical applications since FoxOs plays a critical role in carcinogenesis, while attenuation of FoxOs improves prognosis [57]. Whether prolonged exercise combined with severe energy deficit could elicit FoxOs downregulation in cancer cells remains unknown, but this may be another mechanism by which exercise could have beneficial effects in some cancers [52].

Muscle mass was reduced less in the lower than that of the upper extremities, and this contrasts with the lower inactivation of FoxOs in the former, which argues against a role of UPS in protein breakdown in our experimental conditions. FoxOs, and primarily FoxO1, reduce carbohydrate oxidation by upregulating the transcription of pyruvate dehydrogenase kinase 4 (PDK4) [58], which inhibits the activity of the pyruvate dehydrogenase complex (PDC) and decreases the glycolytic flux [59]. Moreover, FoxOs are known to prioritize fat oxidation by several mechanisms [60]. Nevertheless, although FoxOs activation may reduce carbohydrate utilization at rest and during exercise, our data indicate that fat oxidation can be increased both in muscles of the upper and lower extremities, despite some inactivation of FoxOs, suggesting that other mechanism/s should explain the increase in fat oxidation during a severe energy deficit, such as an increased leptin signaling [37]. The muscles of the upper extremities have a higher capacity for carbohydrate oxidation than the lower extremities during exercise [61], and under FoxOs inactivation enhanced signaling of alternative pathways stimulating fat oxidation is required. In fact, we observed a higher increase in leptin signaling in the deltoid muscles than in vastus lateralis of our subjects [37].

### 4.2. The Changes in Circulating EAA, BCAA, Leucine, and Insulin Are Inversely Associated with the Changes in Autophagy Induction in the Muscles Submitted to a Large Exercise Volume

The inverse linear relationship between the changes in anabolic signals, mostly EAA, BCAA, leucine, and insulin and the induction of autophagy (ratio pSer555 ULK1/total ULK1) in the vastus lateralis but not in the deltoid muscles, could indicate that a high exercise volume may act in concert with anabolic signals to blunt the activation of autophagy. This interpretation is based on the fact that these associations were not observed in the control deltoid or the moderately exercised deltoid. The negative association found between the changes in insulin and the changes in deltoid muscles´ LC3BII/LC3BI ratio (used as a marker of autophagosome formation) may also support a role for insulin in downregulating autophagosome formation. These findings are in partial agreement with the study by Fritzen, et al. [35], which showed in human skeletal muscle that insulin reduces LC3BII/LC3BI ratio and inhibits autophagy induction via ULK1 phosphorylation at Ser757 after an acute exercise bout and after training, Nevertheless, they did not find a differential insulin modulation between the trained and untrained leg in their experimental conditions [35].

Thus, during a severe energy deficit, a large exercise volume may sensitize the skeletal muscle to the anabolic signals that downregulate autophagy induction. While the less exercised muscles are refractory or less sensitive to this effect, even the moderate volume of exercise performed by the trained deltoid resulted in lower total and phosphorylated ULK1. Overall our findings indicate that exercise counteracts the induction of autophagy in skeletal muscle during a severe energy deficit in a dose-dependent fashion and that circulating essential amino acids, and particularly leucine contribute to this effect.

In accordance with the changes in its upstream kinase ULK1, Beclin 1 phosphorylation at Ser15 was largely increased in the deltoid muscles but completely blunted in the vastus lateralis. The phosphorylation of Ser15 Beclin 1 permits the disruption of the Beclin 1-BCL2 complex, an essential step to for completion of autophagosome formation, which would otherwise interrupt the autophagic flux [27]. Thus, our data indicate that the high exercise volume done by the vastus lateralis suppresses the autophagic response, explaining greater preservation of muscle mass in the lower extremities. In contrast, the moderate exercise volume performed by the deltoid muscles was not enough to blunt Ser15 Beclin 1 phosphorylation. This interpretation is further supported by the lack of associations between the changes in anabolic and catabolic hormones and the changes in Beclin 1 phosphorylation in the vastus lateralis, indicating that local contraction-dependent mechanisms of control prevail over the neuroendocrine regulation.

The potential significance of the exercise-mediated modulation of Beclin 1 during a negative energy balance seen in this study should be underscored, as Beclin 1 regulation plays an essential role in tumorigenesis by mechanisms related and unrelated to autophagy [62,63].

### 4.3. The Ubiquitin-Proteasome System Is not Primarily Mediating Skeletal Muscle Protein Degradation during a Severe Energy Deficit

No sign of an upregulation at the protein level of the two principal E3s in response to a severe energy deficit was seen in the present investigation. In agreement, no significant changes were observed in FBXO32/MAFbx gene expression after CRE (1.33-fold and *p* = 0.45) or CD (0.72-fold and *p* = 0.18) [39]. Nonetheless, we previously reported that in the vastus lateralis, following the four-day intervention gene expression of TRIM63/MuRF1,2 was significantly increased (1.6-fold and *p* = 0.01) as well as a large number of genes encoding proteasome-mediated degradation proteins, with these changes reverted after CD [39]. An increased mRNA expression of MuRF1 and MAFbx was reported following 21 days of 40% energy restriction although the activity of the 26S proteasome was reduced [64]. In contrast, with a similar energy deficit during 10 days, mRNA expression of both E3s was unaltered [13], and another study reported no change in MAFbx protein expression following a 20% energy deficit for 10 days, despite a 60% increase in muscle protein breakdown [12]. Conflicting results have been reported regarding the effects of acute bouts of prolonged exercise on the ubiquitin-proteasome system, with increased MuRF1 and MAFbx mRNA expression following a 200 km run but decreased ubiquitin-conjugated proteins (UbCP) and unchanged chymotrypsin-like activity and protein level of the 20S α + β proteasome subunits [32]. The same research group reported a 55% increase in MuRF1 protein expression but unchanged MAFbx and UbCP protein levels following a 24-h run [31].

In agreement with reduced activation of the E3s in the present investigation, the MAFbx downstream target eIF3f did not change with the intervention. Had the activity of MAFbx been increased, eIF3f should have been increasingly ubiquitinated and degraded at the proteasome [65]. In agreement with Kim et al. [32], our results point towards a minor role of UPS in protein breakdown during a large energy deficit eliciting a loss of ~2% to 4% of the skeletal muscle mass. Furthermore, the lower expression of MuRF1 in the deltoid muscles fits well with the enhanced inhibition of FoxOs in that muscle group. Notwithstanding, it should be considered that E3s and, particularly, MuRF1 and MAFbx are auto-ubiquitinated during proteasomal degradation, and therefore increased expression of the transcriptional program during catabolic conditions could be required to avoid a decrease of E3s protein levels [18].

### 4.4. Signaling Data Indicate that the Autophagy-Lysosome-Mediated Protein Degradation Is Attenuated by High-Volume Low-Intensity Exercise, Likely Contributing to the Preservation of Muscle Mass in the Lower Extremities

We found no change in the ratio LC3BII/LC3BI, which is used as a marker of autophagosome formation, as LC3BI is lipidated to form LC3BII during autophagy, which together with the engulfed materials are targeted for lysosomal degradation. A potential upregulation of MAP1LC3B gene expression (1.4-fold and *p* = 0.057) [39] and the increased protein levels of LC3BI are compatible with some stimulation of the protein machinery required for autophagosome formation, which contrasts with the unchanged levels of both LCBII and the ratio LC3BII/I. Nevertheless, interpretation of these markers should be made with caution, as an increased autophagosome content at a certain time point could be due to either an accelerated rate of autophagosome formation or inhibited rate of lysosomal degradation [66]. The expression levels of p62/SQSTM1 have been used as a representative marker of autophagosome clearance, although this has been subjected to criticism [28]. Here we observe an increased p62/SQSTM1 protein expression levels in all muscles but higher in the vastus lateralis (~95%) than in the deltoid muscles (~25%). This is in agreement with the previously reported upregulation of p62/SQSTM1 at the gene level being only present in the vastus lateralis (1.7-fold and Padj < 0.001) [39]. These findings are suggestive of reduced autophagosome clearance, particularly, in the vastus lateralis, and taken together with the unaltered LC3BII/LC3BI ratio, can be suggestive of a blockage in autophagy flux, particularly, in the muscles of the lower extremities as compared to the upper extremities.

In humans, an increase in autophagy induction, but likely blocked autophagic flux, has been reported after prolonged endurance exercise (approximately >2 h) [24,25,67,68,69]. Autophagy is known to promote cellular survival by contributing to maintaining appropriate concentrations of amino acids and energy levels [70]. The duration and degree of energy deficit modulate the response in protein turnover. Acute and short-term (less than seven days) energy deficits provoke an increase in whole-body proteolysis, amino acid oxidation, and nitrogen excretion which are progressively attenuated as the extent of the deficit increases until reaching a plateau [8,71,72,73]. This protein-sparing response elicited by sustained food deprivation was suggested as an adaptive mechanism already during the 1940s from the findings of the Minnesota Starvation Study [74]. The energy deficit elicited in four days in the present investigation (~22,000 kcal) is larger than the accumulated energy deficit in most experiments studying the muscle responses to energy restriction for periods up to 21 days [12,13,64,68].

### 4.5. Skeletal Muscle Seems Unresponsive to the Anticatabolic Effects of EAA during a Severe Energy Deficit

The limited evidence in humans on the effects of amino acids ingestion on MPB indicate that only BCAA or leucine alone can elicit some attenuation of MPB at rest [75,76], and following exercise under energy balance conditions [77,78,79]. Here, under a severe energy deficit, the ingestion of three daily doses of protein (total of 0.8 g/kg body weight/day) had no distinctive effects on protein markers of the UPS or ALS activity. These results are in agreement with other short-term energy deficit studies, in which even with a higher protein dose (1.2, 1.6, and 2.4 g/kg body weight/day) no significant effects were observed in the protein markers of UPS and ALS activity [13,64]. In this study, the levels of circulating BCAA were increased after CRE in the two groups, although to a greater extent in the whey protein group [41]. This may have attenuated the autophagic response to the energy deficit, but had no stimulating effect on protein synthesis signaling [38], altogether resulting in lean mass loss regardless of the whey protein supplementation [38]. The latter agrees with the fact that the effect of BCAA on muscle protein turnover response is highly dependent on the overall endocrine state (i.e., anabolic versus catabolic) [80].

Data on specific molecular markers of UPS have reported that administration of BCAA or leucine alone reduces MuRF1 and MAFbx mRNA expression in myotubes [81] and rat skeletal muscle [82], although not all agree [83]. However, overall results on human skeletal muscle are in line with the unchanged levels seen here in both groups (protein vs. sucrose) and the lack of association between the changes in EAA, BCAA or leucine and the changes in MuRF1, MAFbx, and eIF3f protein expression, indicating that MuRF1 protein levels in skeletal muscle are not affected by ingestion of amino acids [84,85], including EAA [67] and BCAA [86], despite some controversy [87]. Regarding the regulation of ALS markers, the association between the changes in EAA, BCAA, and leucine with the induction of autophagy (increase of the pSer555 ULK1/total ULK1 ratio) in the vastus lateralis but not in the deltoid muscles, may suggest that a large exercise volume can facilitate the anti-catabolic effect of amino acids to blunt the activation of autophagy. This outcome is in line with the elevation of the mTOR:ULK1 complex (a marker of autophagy inhibition) seen in humans after ingesting of a mixed EAA solution with a high-leucine content (∼3.5 g) [78]. Likewise, the same study [78] reported a decreased LC3BII protein expression after ingestion of both moderate (∼1.8 g) and high-leucine solutions (∼3.5 g), which was attributed to the higher increase in insulin in the high-leucine group [35]. The negative association found here between the ratio LC3BII/LCBI from PRE to CRE and from PRE to CD with the changes in insulin may suggest that the low serum insulin concentrations observed in the present investigation prevented the expected inhibition of autophagosome formation by the ingestion of proteins. This is supported by studies in humans showing that a mixed EAA + carbohydrate meal reduces LCBII following resistance exercise [67].

### 4.6. Limitations

The present study does not include a non-exercise caloric restricted control group, because inducing a daily energy deficit of 5500 kcal is not achievable exclusively by fasting. It may be argued that the response to exercise and caloric restriction cannot be compared between deltoid and vastus lateralis muscles due to differences in fiber type composition. Nevertheless, substantial experimental evidence has shown that both muscles adapt similarly to training and immobilization. To date, no single study in human or animal models has assessed whether lower and upper limbs respond differently to a severe energy deficit. Nevertheless, it has been shown that deltoid and vastus lateralis, despite small differences in fiber type composition and protein expression [42], adapt similarly to prolonged low-intensity training [43]. Moreover, despite the triceps brachii presenting a considerably larger proportion of type II fibers than the vastus lateralis [88], both muscles have almost identical functional phenotypes in elite cross-country skiers with similarly highly trained muscles of the upper and lower extremities [89,90].

## 5. Conclusions

The findings of the present investigation indicate that the predominant proteolytic pathway activated in skeletal muscle during a severe energy deficit is the autophagy-lysosomal system and not the ubiquitin-proteasome system. Furthermore, we have demonstrated that the attenuation of the loss of muscle mass elicited by exercise during a severe energy deficit might be due to reduced activation of protein degradation through autophagy, although this muscle-sparing effect does not further benefit from the isolated consumption of protein at RDA doses. Future studies are necessary to determine whether higher protein doses and/or simultaneous consumption of carbohydrate as well as other exercise modes (i.e., resistance exercise or periods of high-intensity exercise) could evoke a greater inhibition of autophagy to elicit higher preservation of lean mass during a severe energy deficit. Finally, the modulation of FoxOs and Beclin 1 by exercise, during a severe energy deficit reported here, identifies novel molecular targets for pharmacological studies aiming to preserve muscle mass in patients with severe energy deficit with incapacity to exercise.

## Figures and Tables

**Table 1:** Characteristics of the overweight and obese male participants included in the study.

Variable	Diet	
	Sucrose	Whey Protein
	(*n* = 7)	(*n* = 8)
Age (years)	38.7 ± 8.2	43.0 ± 8.0
Height (cm)	181 ± 5.5	180 ± 4.2
Body mass (kg)	98 ± 12.0	100 ± 14.9
BMI (kg/m^2^)	29.9 ± 3.1	30.9 ± 4.2
Lean mass (kg)	63.1 ± 3.1	65.4 ± 6.0
Fat mass (kg)	31.5 ± 9.1	31.4 ± 9.2
Body fat (%)	31.6 ± 5.3	30.9 ± 4.1
VO_2_max (L/min)	3.8 ± 0.3	3.9 ± 0.3
Daily energy intake (kcal)	2256 ± 513	2086 ± 489
Physical activity (IPAQ) (kcal/d)	612 ± 315	601 ± 289

BMI, body mass index and IPAQ, International Physical Activity Questionnaire. Data are shown as mean ± SD. Modified from Calbet, et al. [40].

## Supplementary Materials

The following are available online at https://www.mdpi.com/2072-6643/11/11/2824/s1, Figure S1: Correlations between changes in signaling and hormones or amino acids for subjects in the sucrose and whey protein groups. Figure S2: Correlations between changes in signaling and hormones or amino acids from PRE to CRE.
