# Supplementary material for: Exercise Mitigates the Loss of Muscle Mass by Attenuating the Activation of Autophagy during Severe Energy Deficit
Source: Nutrients. 2019 Nov 19;11(11):2824. doi: 10.3390/nu11112824 (PMC6893734; doi:10.3390/nu11112824)
Supplement: Supplementary file 1 [file nutrients-11-02824-s001.pdf]

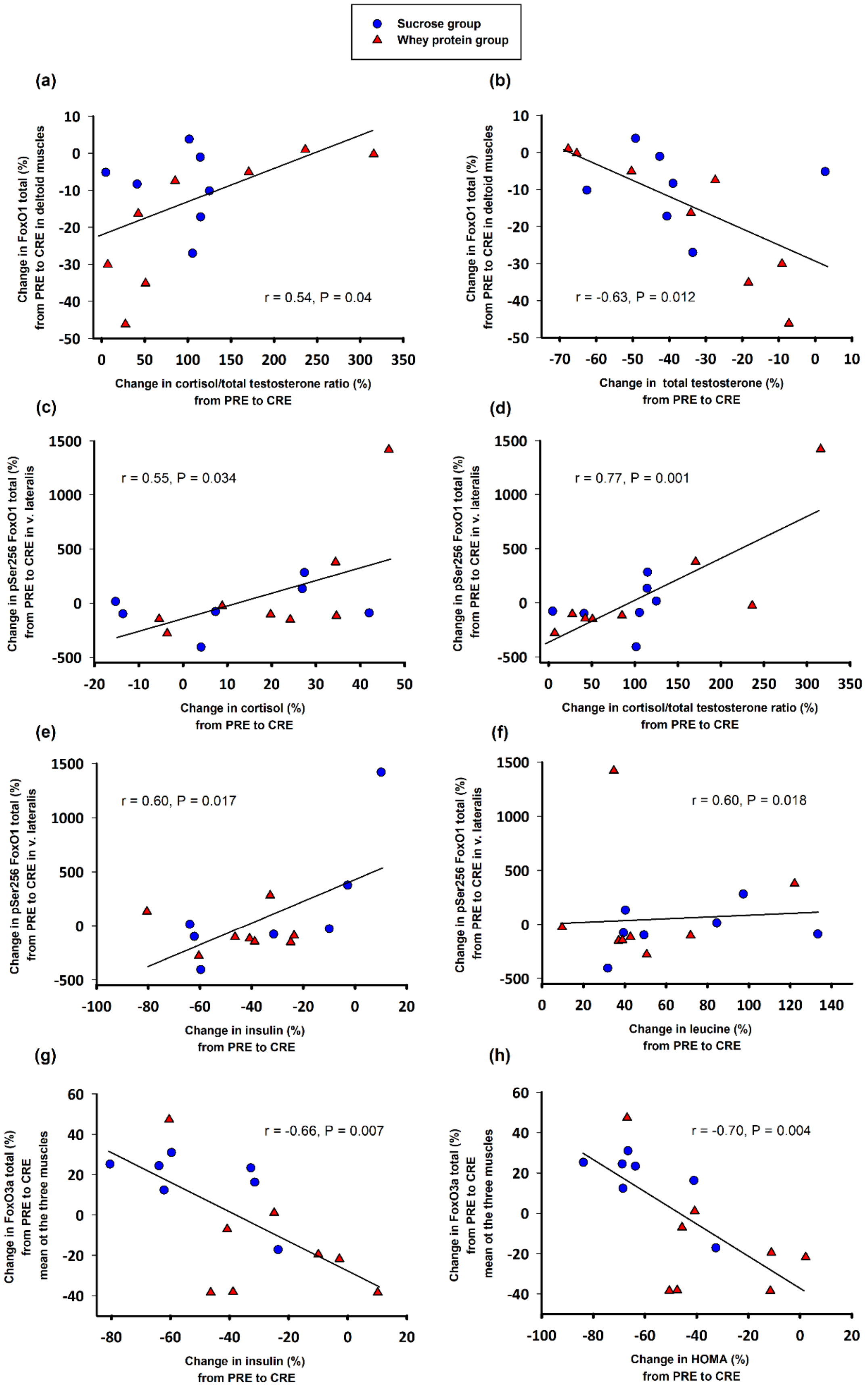

Figure S1. Correlations between changes in signaling and hormones or amino acids for subjects in the sucrose and whey protein groups. *n* = 7 and *n* = 8 for the sucrose and whey protein groups, respectively. Deltoid muscles = mean of both arms.

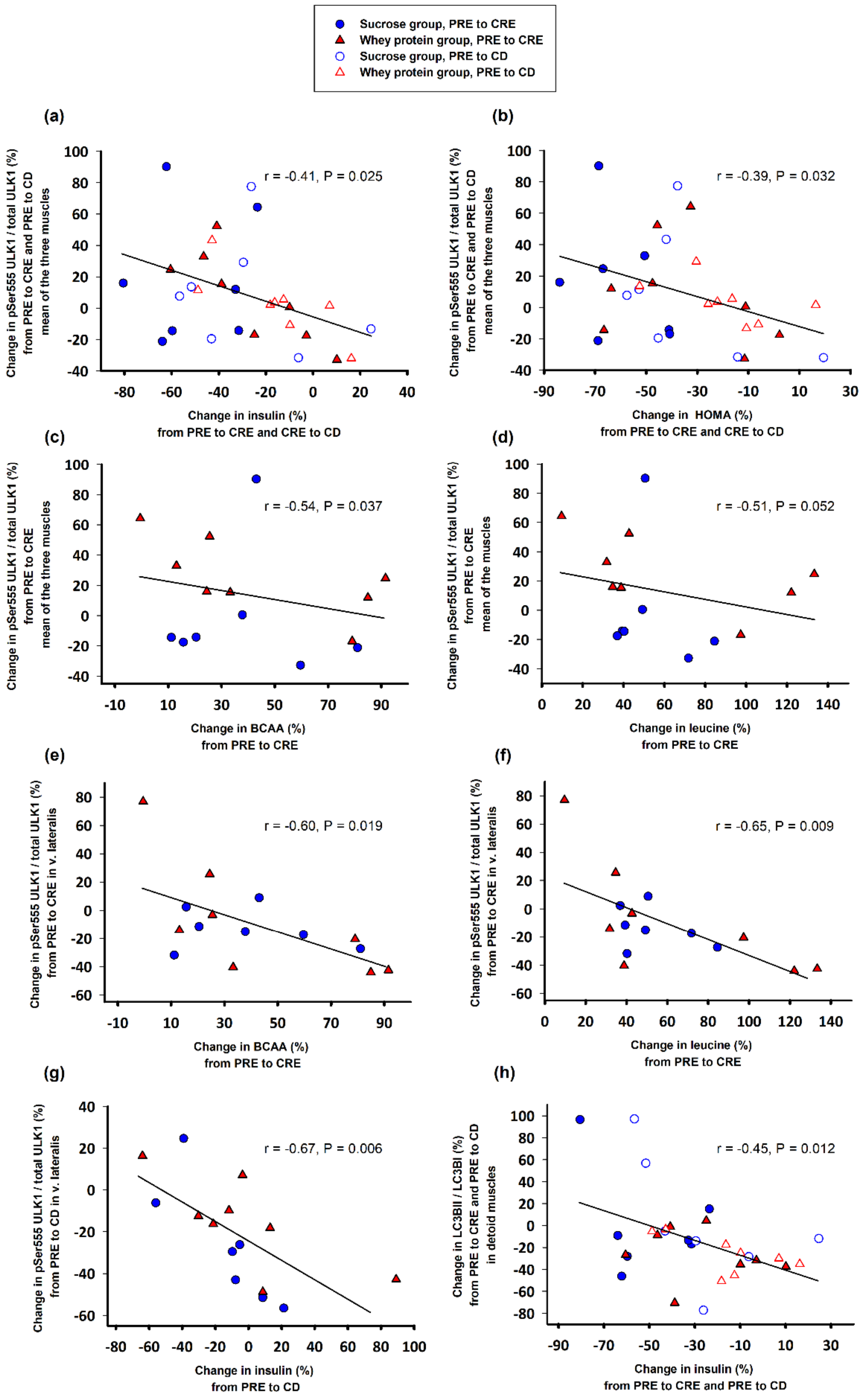

Figure S2. Correlations between changes in signaling and hormones or amino acids from PRE to CRE ($n = 7$ and $n = 8$ for sucrose and whey protein groups, respectively). In a, b, and h correlations from PRE to CRE and PRE to CD are combined ($n = 30$). Deltoid muscles = mean of both arms.
